# Dual antiplatelet therapy in patients with aspirin resistance following coronary artery bypass grafting: study protocol for a randomized controlled trial [NCT01159639]

**DOI:** 10.1186/1745-6215-13-148

**Published:** 2012-08-25

**Authors:** Hrvoje Gasparovic, Mate Petricevic, Tomislav Kopjar, Zeljko Djuric, Lucija Svetina, Bojan Biocina

**Affiliations:** 1 Department of Cardiac Surgery, University Hospital Center Zagreb, University of Zagreb, Kispaticeva 12, 10 000 Zagreb, Croatia

**Keywords:** Dual antiplatelet therapy, Coronary artery bypass grafting, Aspirin resistance

## Abstract

**Background:**

Coronary artery disease remains the dominant cause of mortality in developed countries. While platelets have been recognized to play a pivotal role in atherothrombosis, the ideal antiplatelet regime after coronary artery surgery remains elusive.

The evolution of CABG has presently moved beyond technical improvements to involve modulation of pharmacologic management designed to improve patient outcomes. The aim of this trial will be to test the hypothesis that the addition of clopidogrel to patients with documented postoperative aspirin resistance will reduce the incidence of major cardiovascular events.

**Methods:**

Patients scheduled for isolated coronary artery surgery will be eligible for the study. Patients in whom postoperative multiple electrode aggregometry documents aspirin resistance will be randomized into two groups. The control group will receive 300 mg of aspirin. The dual antiplatelet group will receive 75 mg of clopidogrel in addition to 300 mg of aspirin. Patients will be followed for 6 months. Major adverse cardiac and cerebrovascular events (death from any cause, myocardial infarction, stroke, hospitalization due to cardiovascular pathology) as well as bleeding events will be recorded.

**Discussion:**

This will be the first trial that will specifically address the issue of dual antiplatelet therapy in patients undergoing coronary artery surgery who have been found to be aspirin resistant. In the event that the addition of clopidogrel proves to be beneficial in this subset of surgical patients, this study could significantly impact their future antiplatelet management.

This randomized controlled trial has been registered at the ClinicalTrials.gov website (Identifier NCT01159639).

## Background

Coronary artery disease is a health care issue of epidemic proportions, and has a profound impact on resource utilization. A quiescent atherosclerotic lesion may follow the course of progressive luminal encroachment, or succumb to an acute thrombotic event. Reduced *de novo* collagen synthesis and increased extracellular matrix metabolism contribute to weakening of the fibrous cap [1]. Platelet aggregation is a crucial component of this process. Coronary artery bypass grafting (CABG) remains the best management option for patients with severe multi-vessel coronary artery disease [2]]. It has been subjected to an unparalleled level of scientific scrutiny. The continuous trend toward improving patient outcomes following CABG stems from refinement of the technical aspects of the procedure, as well as optimization of the adjuvant pharmacologic therapy.

### Antiplatelet management following CABG

Platelet inhibition is paramount in the management of coronary artery disease. Antiplatelet drugs reduce mortality and the incidence of major vascular events in patients with a wide variety of vascular occlusive pathologies [3]. Their beneficial effects must be, however, balanced against the associated risk of bleeding. Acetylsalicylic acid (ASA) is currently recommended after CABG in order to improve long-term graft patency [4]]. The recommended doses range from 150 to 325 mg, with a trend towards clinical benefit when higher doses are utilized within the first year [4]]. There is no compelling evidence to support the superiority of clopidogrel to ASA monotherapy in optimizing graft patency following CABG [4]]. The mechanisms of action of thienopyridines and ASA differ, allowing for a cumulative anti-aggregative effect [3,5]. ASA irreversibly suppresses cyclooxygenase-1 activity thereby reducing thromboxane A2 production, whereas clopidogrel acts on the P2Y12 adenosine diphosphate (ADP) receptor to inhibit ADP-mediated platelet aggregation [6,7]. While dual antiplatelet therapy offers a reduction in atherosclerotic events in patients undergoing percutaneous coronary interventions, this benefit has not been reliably reproduced in other clinical settings [7,8]].

### Individual variability to antiplatelet agents

The incidence of interpatient variability to antiplatelet drugs depends upon the laboratory evaluation used to diagnose it [7]. Novel tools for quantifying drug induced platelet inhibition have brought into focus the individual variations in antiplatelet responses. This underscores the importance of identifying the optimal antiplatelet drug protocol in order to achieve the targeted level of anti-aggregation. Multiple electrode aggregometry (MEA) has been shown to be a useful instrument in the quantification of platelet inhibition by ASA [6]. Its mechanism of assessing platelet inhibition is based on changes in impedance on its sensor wires secondary to platelet adherence. Aspirin resistance has been suggested to range anywhere from 1 to 45% [7]. The efficacy of ASA absorption, TXA2 independent platelet activation pathways, COX-1 gene mutations, as well as ASA interactions with other medications have all been postulated to play a role in inducing aspirin resistance [9]. The response to ASA should best be viewed as a continuous variable. Dichotomizing patients into responders or non-responders is, however, suitable for scientific research and is commonly employed for this purpose [7]. The presence of ASA low-responsiveness has been linked to an increase in cardiovascular morbidity [9]. The clinical correlation of individual patient refractoriness to ASA with an increased incidence of cardiovascular complications is fundamental to the present study. This will be, to the best of our knowledge, the first prospective randomized study that will aim to evaluate whether the combination of ASA and clopidogrel in ASA-resistant CABG patients offers a clinical benefit. Our hypothesis that a dual antiplatelet regime will improve patient outcomes in this setting stems from the convergence of the clinical impact of ASA resistance and the benefit gained from adding clopidogrel to ASA in certain clinical scenarios.

## Methods

### Study population

All consecutive patients aged 18 years or more, scheduled for an elective surgical myocardial revascularization procedure using cardiopulmonary bypass over the study period, at the University Hospital Center Zagreb, will be screened for the study. Written informed consent will be obtained from all patients prior to enrolment into the study. The preoperative exclusion criteria will include valvular pathology warranting surgical correction, critical preoperative state, inability to provide informed consent, reoperation, any medical condition for which dual antiplatelet therapy is indicated, redo-CABG, off-pump CABG, preoperative antiplatelet therapy other than ASA or clopidogrel, preoperative anticoagulation with coumadin and history of cerebrovascular accidents. Furthermore, patients who require postoperative anticoagulation, intra-aortic balloon pump support, or who die prior to postoperative day 4 will not be included into the study. All other consecutive isolated CABG patients will be screened for the study. Those patients who exhibit aspirin resistance on their MEA evaluation on postoperative day 4 will then be randomized into two groups.

### Ethics

The Ethics Committee of the University Hospital Center Zagreb has evaluated and approved the conduct of this study (No. 01/001/VG) on 23 June 2010. Ethical standards in line with the Declaration of Helsinki involving research on human subjects will be strictly adhered to. This protocol complies with the CONSORT guidelines [10].

### Sample size

An exact binomial test power analysis was performed in order to estimate the approximate sample size required to test the null hypothesis. The minimum effect size was 10%. Accounting for a maximum estimated 10% loss to follow-up, a total of 219 patients will be required to test the null hypothesis with an α value of 0.05 and a power of 0.8.

### Aspirin low-responsiveness

The ASPI test evaluates cyclooxygenase-dependent platelet aggregation (using arachidonic acid) that is sensitive to aspirin. Our definition of low response to ASA will be based upon our own published experience with multiple electrode aggregometry in a similar population of patients [11]. In brief, patients in the aforementioned study were divided into quartiles with respect to their ASA responsiveness, and those in whom the ASPI test was greater than the 75^th^ percentile of the entire cohort (that is, area under the curve, AUC ≥ 30 units) were defined as being ASA low-responders [11]. This definition has been corroborated by published data from other sources [12-14].

### Randomization of patients and interventional strategy

**Figure 1:**
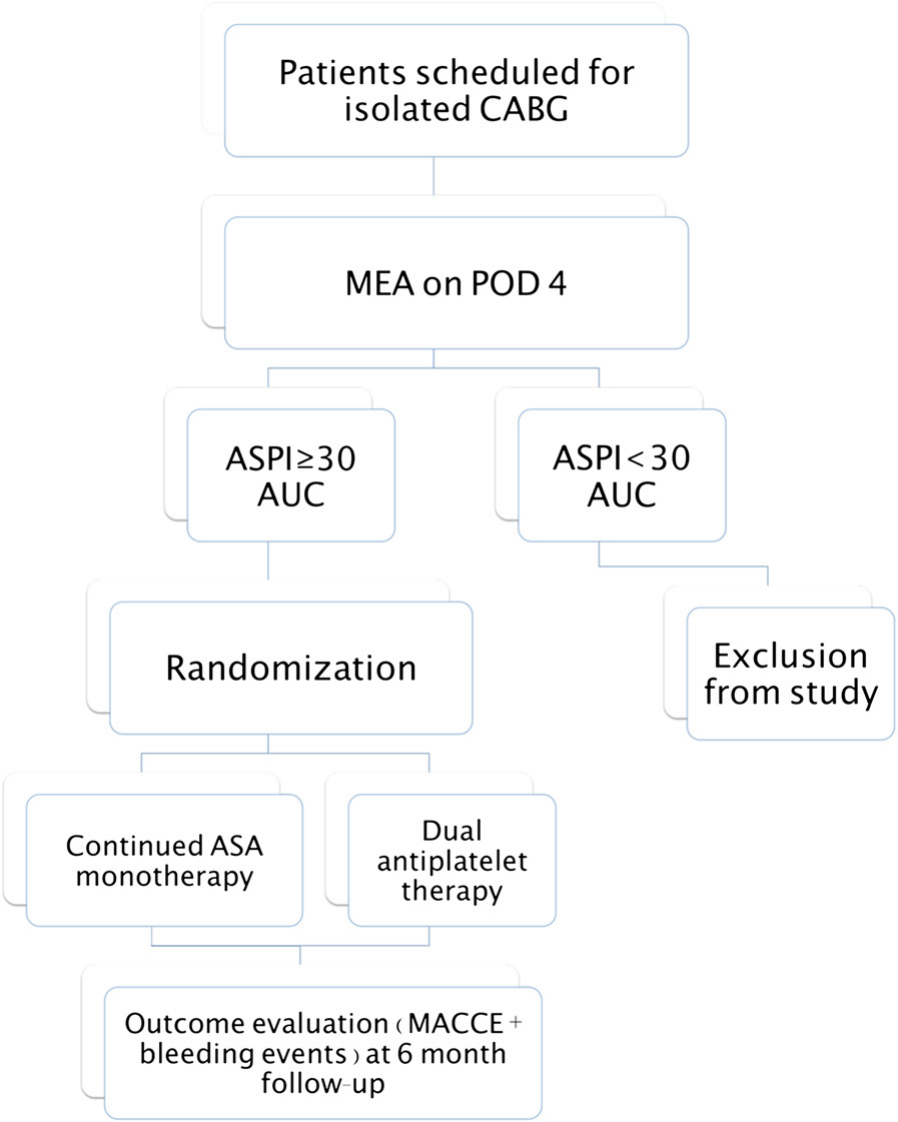
**Flowchart detailing the screening algorithm for patient recruitment, followed by subsequent randomization into the aspirin monotherapy or dual antiplatelet therapy groups.** CABG, coronary artery bypass grafting; MEA, multiple electrode aggregometry; ASPI, cyclooxygenase dependent platelet aggregation, AUC, area under the curve; ASA, acetylsalicylic acid; MACCE, major adverse cardiac and cerebrovascular events.

All post-CABG patients in whom none of the postoperative exclusion criteria have occurred will undergo MEA analysis on postoperative day 4. Patients found to have evidence of ASA low-responsiveness, as defined by an ASPI test ≥ 30 AUC, will undergo randomization into either the control group or the dual antiplatelet therapy (dAPT) group. Assignment to either group will be performed by random allocation using randomization software [15]. Patients in the control group will continue to receive 300 mg of ASA in accordance to our hospital’s standard post-CABG antiplatelet management strategy. Patients in the dAPT group will, conversely, be subjected to augmentation of their antiplatelet regime by receiving 75 mg of clopidogrel in addition to the 300 mg of ASA. A flowchart depicting the screening and randomization protocols is shown in Figure 1.

### Preoperative antiplatelet therapy management

Preoperative antiplatelet therapy is managed by the referring cardiologist. It almost uniformly consists of 100 mg of ASA and this is continued up to the day of surgery. Some patients receive clopidogrel in addition to ASA in the preoperative period. Clopidogrel will be discontinued 5 days prior to the scheduled revascularization procedure. All patients included in the study will have received antiplatelet therapy for no less than 14 days prior to enrolment.

### Perioperative management

The perioperative management of study patients will not deviate from our hospital’s standard preoperative anesthetic regime, which includes diazepam and morphine prior to induction of anesthesia. Endotracheal tube, urinary catheter, radial artery and pulmonary artery catheters will be inserted. Our protocol will consist of induction and maintenance of anesthesia with midazolam, fentanyl and pancuronium bromide. This will be complemented with sevoflurane inhalation. The initial ventilator settings will consist of a tidal volume of 8 ml/kg, and a respiratory rate of 12 breaths per minute. Systemic heparinization with a target-activated clotting time > 480 seconds will be utilized, followed by full reversal with protamine after decannulation. The cardiopulmonary circuit will include the Medtronic Affinity Trillium membrane oxygenator, venous reservoir and PVC tubing (Medtronic, Minneapolis, USA) and a Stoeckert III roller pump (Stoeckert, Munich, Germany). The ascending aorta and right atrium will be cannulated for cardiopulmonary bypass (CPB). The CPB flow will approximate 2.4 L/min/m^2^. Every effort will be made to maintain the mean systemic pressure during CPB between 60 and 80 mmHg. Vasopressors will be used as needed in order to achieve the target mean pressure. The patient’s temperature will be allowed to drift down to 32 to 34°C. Myocardial protection will be based upon a combination of antegrade and retrograde cardioplegia, coupled with topical cooling. Both the coronary and the proximal aortic anastomoses will be performed on an arrested heart, during a single period of aortic cross-clamping. Weaning from CPB will typically be performed without inotropic support. In patients in whom the cardiac index will remain below 2.2 L/min/m^2^, dobutamine will be administered. Norepinephrine will be instituted if dobutamine produces excessive vasodilatation. Escalation of vasoactive support, if necessary, will include the addition of another inotrope, an intra-aortic balloon pump or mechanical circulatory assistance.

Postoperative medications will typically include a beta blocker, a hydroxy-methyl-glutaryl-CoA reductase inhibitor and a diuretic. Electrolytes will be supplemented as indicated. An ACE inhibitor will be instituted, as tolerated. Peptic ulcer prophylaxis will be universally implemented, and continued for six weeks postoperatively. Non-steroidal anti-inflammatory drugs will generally not be administered in the early postoperative period. The antiplatelet regime will be based upon the previously mentioned randomization protocol.

### Blood sampling

Blood samples for MEA measurements will be obtained on postoperative day (POD) 4 via peripheral venipuncture. The search for an ideal anticoagulant for blood samples used for the quantification of platelet function is still ongoing. Heparin will be the anticoagulant used in the present study [16,17]. Blood will be collected in 4 ml heparin (Lithium Heparin 68 IU)-coated BD Vacutainer plastic tubes. Routine laboratory evaluations will be performed and recorded preoperatively and on POD 4. The same person, who has no direct involvement in either patient care or postoperative follow-up, will perform all MEA measurements. Blood samples will be allowed to rest for 30 minutes after blood withdrawal and MEA will be performed subsequently.

### Multiple electrode aggregometry (MEA)

Whole blood aggregability will be determined using a new generation impedance aggregometer (Multiplate® analyzer, Dynabyte Medical, Munich, Germany). The method has been described in detail previously, and has already been validated [18]. In brief, the principle underlying MEA is that platelets are non-thrombogenic in their resting state, but have the potential to present multiple surface receptors when activated, which enables them to attach to a variety of surfaces. The accumulation of platelets on the Multiplate sensor wires will increase the electrical resistance between them. This variability in impedance, which is expressed in arbitrary AUC units, is the MEA parameter with the highest diagnostic value [18,19]. The analysis takes 3 minutes for incubation and 6 minutes for the post-stimulation measurement. Platelet aggregation was determined in response to stimulation with arachidonic acid with a final concentration of 0.5 mM (ASPI test designed to evaluate ASA effect) and ADP with a final concentration of 6.4 μM (ADP test designed to evaluate thienopyridines, that is, clopidogrel).

### Patient follow-up

Prospective collection of the follow-up data will be carried out postoperatively via three bimonthly telephone interviews over the initial 6 months. Three surgical residents will perform the acquisition of the follow-up data. The telephone interview will be based upon a customized questionnaire, which incorporates information critical to the present study. Follow-up data will then be stored in a central database. Patients in whom the interview revealed equivocal information pertinent to the study will be asked to consign their medical records for further review by a cardiac surgeon and a cardiologist blinded to the study protocol. In the case that the opinions of the two reviewers differ, a third reviewer will offer an opinion, which will then be considered definitive and will be stored in the central database.

### Outcome definitions

The primary clinical endpoint of the study will be the incidence of major adverse cardiac and cerebrovascular events (MACCE) in relation to two distinct antiplatelet strategies (ASA monotherapy or dual antiplatelet therapy) in patients found to be ASA-resistant. MACCE will be defined as a composite endpoint consisting of all-cause mortality, non-fatal myocardial infarction, cerebrovascular accident and cardiovascular rehospitalization. The definition of perioperative and postoperative myocardial infarction will be based upon published recommendations of the Joint European Society of Cardiology/American College of Cardiology Foundation/American Heart Association/World Heart Federation Task Force for the Redefinition of Myocardial Infarction [20]. The definition of stroke will be based upon a new onset focal neurological deficit lasting > 24 hours or an imaging study suggestive of an acute clinically relevant cerebral lesion in patients with rapid recovery [21].

Bleeding events will constitute the secondary clinical endpoint. They will be stratified in accordance to the consensus report from the Bleeding Academic Research Consortium (BARC). In brief, the hemorrhagic complications according to the BARC scale range from type 1, which denotes bleeding that is not actionable, to type 5 which is defined as a bleeding-related fatal event [22]. Type 0 denotes the absence of bleeding [22].

### Statistical analysis

The continuous data will be presented as mean values ± standard deviation (SD) or medians with their respective interquartile range. Categorical variables will be presented as absolute numbers with percentages. Analyses of continuous data between different groups of patients will be performed using the Mann-Whitney *U*-test. Differences between categorical variables will be evaluated with Fisher’s exact test. A two-tailed *P*-value will be used amd *P* < 0.05 will be considered significant for all deployed statistical calculations. The data will be processed using the Statistica 9 software package (StatSoft Inc., Tulsa, USA).

## Discussion

The disruption of the integrity of atherosclerotic lesions followed by secondary thrombosis is recognized as the primary lesion responsible for a variety of acute ischemic syndromes [9]. Continued improvement of surgical outcomes in patients with coronary artery disease is the result of a convergence of technical progress and better tailoring of pharmacotherapy. The fundamental goal of this study is to test the hypothesis that tailored modulation of platelet activity based on reproducible laboratory measurements of antiplatelet drug responses has the potential for improving patient outcomes following CABG. Patients with documented aspirin resistance early after CABG will be randomized to receive either additional clopidogrel or no augmentation of their anti-aggregation management. The incidence of MACCE will be recorded, as well as the safety profile in terms of bleeding events for both treatment arms.

The design of this study is a prospective randomized one. It will enrol consecutive cardiac surgical patients based upon pre-specified criteria. The discriminatory variable among the screened patients will be a reproducibly quantifiable deficiency in their antiplatelet therapy response. This will create a homogenous patient population, allowing for a robust analysis. We believe that the adverse impact of aspirin resistance on patient outcomes coupled with its relative frequency add merit to a study such as this. It is paramount, however, to objectively weigh the hypothetical benefit of dual antiplatelet therapy against the potentially increased susceptibility for bleeding. The benefit of dual antiplatelet therapy has not been universally reproducible in all publications dealing with the subject. Kulik *et al*. have evaluated the effect of adding clopidogrel to ASA on the degree of intimal hyperplasia in saphenous vein coronary artery bypass grafts [23]. No significant reduction in intimal hyperplasia was observed in that study [23]. Conversely, Gao and coauthors documented superior venous graft patency with dual antiplatelet therapy in comparison to ASA monotherapy [24]. Neither of these studies explored the impact of interpatient variability to antiplatelet drug therapy on patient outcomes. The strategy of adding a thienopyridine to patients who have been found to be ASA resistant following CABG has thus far been unexplored. The present trial is designed to offer more information on that specific subject.

Should the results of this study prove to document superiority of the dual antiplatelet regime in patients found to be aspirin resistant, this could promote an alteration of the contemporary management of platelet inhibition in patients undergoing CABG towards a more aggressive approach.

## Trial status

Patient recruitment for the study is currently ongoing.

## Abbreviations

ADP: Adenosine diphosphate; ASA: Acetylsalicylic acid; ASPI: Cyclooxygenase-dependent platelet aggregation; AUC: Area under the curve; CABG: Coronary artery bypass grafting; CPB: Cardiopulmonary bypass; dAPT: Dual antiplatelet therapy; MACCE: Major adverse cardiac and cerebrovascular events; MEA: Multiple electrode aggregometry; POD: Postoperative day.

## Competing interests

The authors have no competing interests to declare.

## Authors’ contributions

HG is the principle investigator. He participated in designing the study, defining the study protocol and also drafted the manuscript. MP helped design the study as well as the study protocol. He contributed to drafting the manuscript. Additionally, he will be involved in acquiring data pertaining to the multiple electrode aggregometry. TK helped design the study and the study protocol. Additionally, he will be involved in obtaining and collecting follow-up information. He also contributed to drafting the manuscript. ZD helped design the study protocol, and will participate in gathering follow up information. LS will participate in obtaining and collecting follow-up information. BB revised the manuscript for critically important content and will coordinate the statistical analysis. All authors read and approved the final manuscript.

## References

[B1] Libby P Bonow RO, Mann DLm Zipes DP, Libby P Braunwald’s heart disease: A textbook of cardiovascular medicine The vascular biology of atherosclerosis 2002 9 Philadelphia, PA: Elsevier Saunders 897 913

[B2] Taggart DP Altman DG Gray AM Lees B Nugara F Yu LM Campbell H Flather M ART Investigators. Randomized trial to compare bilateral vs. single internal mammary coronary artery bypass grafting: 1-year results of the Arterial Revascularisation Trial (ART) Eur Heart J 2010 31 2470 2481 10.1093/eurheartj/ehq318 20805116

[B3] Antithrombotic Trialists’ Collaboration Collaborative meta-analysis of randomised trials of antiplatelet therapy for prevention of death, myocardial infarction, and stroke in high risk patients BMJ 2002 324 71 86 10.1136/bmj.324.7329.71 11786451 PMC64503

[B4] Dunning J Versteegh M Fabbri A Pavie A Kolh P Lockowandt U Nashef SA EACTS Audit and Guidelines Committee. Guideline on antiplatelet and anticoagulation management in cardiac surgery Eur J Cardiothorac Surg 2008 34 73 92 10.1016/j.ejcts.2008.02.024 18375137

[B5] Bollati M Gaita F Anselmino M Antiplatelet combinations for prevention of atherothrombotic events Vasc Health Risk Manag 2011 7 23 30 21339910 10.2147/VHRM.S12271 PMC3037086

[B6] Mengistu AM Mayer J Boldt J Röhm KD Suttner SW Usefulness of monitoring platelet function by multiple electrode aggregometry in primary coronary artery bypass surgery J Cardiothorac Vasc Anesth 2011 25 42 10.1053/j.jvca.2010.02.008 20427204

[B7] Ben-Dor I Kleiman NS Lev E Assessment, mechanisms, and clinical implication of variability in platelet response to aspirin and clopidogrel therapy Am J Cardiol 2009 104 227 233 10.1016/j.amjcard.2009.03.022 19576352

[B8] Fintel DJ Antiplatelet therapy in cerebrovascular disease: implications of Management of Artherothrombosis with Clopidogrel in High-risk Patients and the Clopidogrel for High Artherothrombotic Risk and Ischemic Stabilization, Management, and Avoidance studies’ results for cardiologists Clin Cardiol 2007 30 604 614 10.1002/clc.20154 17847044 PMC6652940

[B9] Kasotakis G Pipinos II Lynch TG Current evidence and clinical implications of aspirin resistance J Vasc Surg 2009 50 1500 1510 10.1016/j.jvs.2009.06.023 19679423

[B10] Schulz KF Altman DG Moher D CONSORT Group CONSORT 2010 Statement: updated guidelines for reporting parallel group randomised trials Trials 2010 11 32 10.1186/1745-6215-11-32 20334632 PMC2857832

[B11] Petricevic MBB Konosic S Kopjar T Milosevic M Gasparovic H Forum THS Definition of acetylsalicylic acid resistance using multiple electrode aggregometry in patients following coronary artery bypass grafting21st World Congress World Society of Cardio-Thoracic Surgeons 2011 Berlin, Germany: Abstract book 70 71

[B12] Skoric B Milicic D Lovric D Gornik I Skoric KN Sertic J Initial patency of the infarct-related artery in patients with acute ST elevation myocardial infarction is related to platelet response to aspirin Int J Cardiol 2010 140 356 358 10.1016/j.ijcard.2008.11.031 19046612

[B13] Jambor C Weber CF Gerhardt K Dietrich W Spannagl M Heindl B Zwissler B Whole blood multiple electrode aggregometry is a reliable point-of-care test of aspirin-induced platelet dysfunction Anesth Analg 2009 109 25 31 10.1213/ane.0b013e3181a27d10 19439684

[B14] von Pape KW Dzijan-Horn M Bohner J Spannagl M Weisser H Calatzis A Control of aspirin effect in chronic cardiovascular patients using two whole blood platelet function assays. PFA-100 and Multiplate Hämostaseologie 2007 27 155 160 10.1007/978-3-540-36715-4_49 17694222

[B15] Research randomizerhttp://www.randomizer.org

[B16] Kalb ML Potura L Scharbert G Kozek-Langenecker SA The effect of ex vivo anticoagulants on whole blood platelet aggregation Platelets 2009 20 7 11 10.1080/09537100802364076 19172515

[B17] Truss NJ Armstrong PC Liverani E Vojnovic I Warner TD Heparin but not citrate anticoagulation of blood preserves platelet function for prolonged periods J Thromb Haemost J Thromb Haemost 2009 7 1897 1905 19694945 10.1111/j.1538-7836.2009.03589.x

[B18] Toth O Calatzis A Penz S Losonczy H Siess W Multiple electrode aggregometry: a new device to measure platelet aggregation in whole blood Thromb Haemost 2006 96 781 788 17139373

[B19] Calatzis A Krueger WB A new approach to platelet function analysis in whole blood- the multiplate analyzer Platelets 2004 15 479 51715763889

[B20] Thygesen K Alpert JS White HD on behalf of the Joint ESC/ACCF/AHA/WHF Task Force for the Redefinition of Myocardial Infarction Universal definition of myocardial infarction Circulation 2007 116 2634 2653 10.1161/CIRCULATIONAHA.107.187397 17951284

[B21] Sacco RL Adams R Albers G Alberts MJ Benavente O Furie K Goldstein LB Gorelick P Halperin J Harbaugh R Johnston SC Katzan I Kelly-Hayes M Kenton EJ Marks M Schwamm LH Tomsick T American Heart Association; American Stroke Association Council on Stroke; Council on Cardiovascular Radiology and Intervention; American Academy of Neurology. Guidelines for prevention of stroke in patients with ischemic stroke or transient ischemic attack: a statement for healthcare professionals from the American Heart Association/American Stroke Association Council on Stroke: co-sponsored by the Council on Cardiovascular Radiology and Intervention: the American Academy of Neurology affirms the value of this guideline Stroke 2006 37 577 617 10.1161/01.STR.0000199147.30016.74 16432246

[B22] Mehran R Rao SV Bhatt DL Gibson CM Caixeta A Eikelboom J Kaul S Wiviott SD Menon V Nikolsky E Serebruany V Valgimigli M Vranckx P Taggart D Sabik JF Cutlip DE Krucoff MW Ohman EM Steg PG White H Standardized bleeding definitions for cardiovascular clinical trials: a consensus report from the Bleeding Academic Research Consortium Circulation 2011 123 2736 2747 10.1161/CIRCULATIONAHA.110.009449 21670242

[B23] Kulik A Le May MR Voisine P Tardif JC Delarochelliere R Naidoo S Wells GA Mesana TG Ruel M Aspirin plus clopidogrel versus aspirin alone after coronary artery bypass grafting: the clopidogrel after surgery for coronary artery disease (CASCADE) Trial Circulation 2010 122 2680 2687 10.1161/CIRCULATIONAHA.110.978007 21135365

[B24] Gao G Zheng Z Pi Y Lu B Lu J Hu S Aspirin plus clopidogrel therapy increases early venous graft patency after coronary artery bypass surgery a single-center, randomized, controlled trial J Am Coll Cardiol 2010 9 56 1639 1643 21050973 10.1016/j.jacc.2010.03.104

